# Symbiont‐conferred reproduction and fitness benefits can favour their host occurrence

**DOI:** 10.1002/ece3.3784

**Published:** 2018-01-03

**Authors:** Yan‐Kai Zhang, Kun Yang, Yu‐Xi Zhu, Xiao‐Yue Hong

**Affiliations:** ^1^ Department of Entomology Nanjing Agricultural University Nanjing China; ^2^ College of Life Sciences Hebei Normal University Shijiazhuang China

**Keywords:** double infection, fitness, reproduction, symbiont, *Tetranychus truncatus*

## Abstract

Double infections of *Wolbachia* and *Spiroplasma* are frequent in natural populations of *Tetranychus truncatus*, a polyphagous mite species that has been a dominant species in China since 2009. However, little is known about the causes and ecological importance of such coexistences. In this study, we established *T. truncatus* strains with different infection types and then inferred the impact of the two endosymbionts on host reproduction and fitness. Double infection induced cytoplasmic incompatibility, which was demonstrated by reduction in egg hatchability of incompatible crosses. However, doubly infected females produced more eggs relative to other strains. *Wolbachia* and *Spiroplasma* did not affect host survival, whereas doubly infected females and males developed faster than other strains. Such reproduction and fitness benefits provided by double infections may be associated with the lower densities of each symbiont, and the quantitative results also confirmed competition between *Wolbachia* and *Spiroplasma* in doubly infected females. These symbiont‐conferred beneficial effects maintain stable prevalence of the symbionts and also help drive *T. truncatus* outbreaks in combination with other environmental factors.

## 1 INTRODUCTION

Heritable bacterial endosymbionts are widespread in arthropods. They utilize diverse strategies to spread within host populations, such as provision of essential nutrients (Gündüz & Douglas, 2009; Hosokawa, Koga, Kikuchi, Meng, & Fukatsu, 2010), defense against parasites (Hedges, Brownlie, O'Neill, & Johnson, 2008; Vorburger, Gehrer, & Rodriguez, 2010), and reproductive manipulation (Ma, Vavre, & Beukeboom, 2014).

Endosymbionts have traditionally been grouped into primary and secondary symbionts. Primary symbionts are mutualists that tend to have a nutritional value, which is the case in the typical example of aphids and *Buchnera aphidicola* (Baumann, 2005). In contrast, secondary symbionts are often facultative symbionts from the host's perspective and have a much broader array of effects. Most symbionts are exclusively or predominantly vertically transmitted; however, the often‐found incongruence between host and symbiont phylogenies indicates that these infections are generally short‐lived on macro‐evolutionary timescales. In theory, once a symbiont invades a novel host, it must immediately be capable of replication and colonization of the female germ line. Hurst and Darby (2009) also speculated that high rates of horizontal transmission, reproductive manipulation, and direct fitness benefits to the host will favour maintenance of secondary symbionts within the host population. The high prevalence of several secondary symbionts without obvious parasitic phenotypes can sometimes be linked to such direct fitness effects; for example, increased performance and sex ratio bias of infected whiteflies sufficiently explain the spread of *Rickettsia* symbionts (Himler et al., 2011).

Among the secondary symbionts, *Wolbachia* is by far the most widely documented and infects a large proportion of arthropod species (Werren, Baldo, & Clark, 2008). *Wolbachia* is notorious for its reproductive parasitism, which ensures its spread despite lowering host fitness. However, even for reproductive parasites, it can be beneficial to enhance host fitness. Indeed, recent years have seen a growing body of evidence on *Wolbachia*‐associated fitness benefits (reviewed in Zug & Hammerstein, 2015). *Spiroplasma* is a wall‐less bacterium associated with diverse arthropods and plants in which it has commensal, pathogenic, or mutualistic effects (Cisak et al., 2015). Some *Spiroplasma* species and strains are known as male killers in fruit flies, ladybird beetles, planthoppers, and butterflies, wherein infected females produce all‐female or female‐biased offspring (Jiggins, Hurst, Jiggins, VD Schulenburg, & Majerus, 2000; Sanada‐Morimura, Matsumura, & Noda, 2013; Tinsley & Majerus, 2006; Williamson et al., 1999). Independent studies have shown that the two symbionts simultaneously infect some arthropods (Duron et al., 2008; Enigl & Schausberger, 2007; Goodacre, Martin, Thomas, & Hewitt, 2006; Jaenike, Stahlhut, Boelio, & Unckless, 2010; Shokal et al., 2016). In *Drosophila neotestacea*, recent spread and mutualism account for the positive association between *Wolbachia* and *Spiroplasma* (Jaenike et al., 2010). However, the ecological and evolutionary importance of such coexistences remains underexplored.

*Tetranychus truncatus* is a polyphagous species that infests more than 60 host plant species (Bolland, Gutierrez, & Flechtmann, 1998). It is mainly distributed in East and Southeast Asia, and there is report of its presence in the USA (Migeon & Dorkeld, 2006, 1996). *T. truncatus* have already become the dominant pest in China since 2009 based on analysis of spider mite sampling data from 2002 to 2015. *T. truncatus* are host to several symbionts; for example, previous studies revealed that *T*. *truncatus* is infected with diverse *Wolbachia* strains (Zhang et al., 2013), and double infections of *Wolbachia* and *Cardinium* are frequent in some natural populations (Zhao, Zhang, & Hong, 2013). Recently, high prevalence of *Spiroplasma* was identified in *T. truncatus*, and there was a substantial positive association between *Wolbachia* and *Spiroplasma* in some populations (Zhang, Chen, Yang, Qiao, & Hong, 2016).

Given the high rate of co‐transmission of these two symbionts and recent *T. truncatus* outbreaks, we investigated the mutualistic phenotypes of these two symbionts within *T. truncatus*. We established different *T. truncatus* strains with the same host genetic background and investigated the effect of *Wolbachia* alone or in combination with *Spiroplasma* on *T. truncatus* reproduction and fitness. Furthermore, the densities of the two symbionts were monitored and compared during host development to infer their competition relationship.

## 2 MATERIALS AND METHODS

### 2.1 *Wolbachia* and *Spiroplasma* prevalence in *T. truncatus*

**Figure 1:**
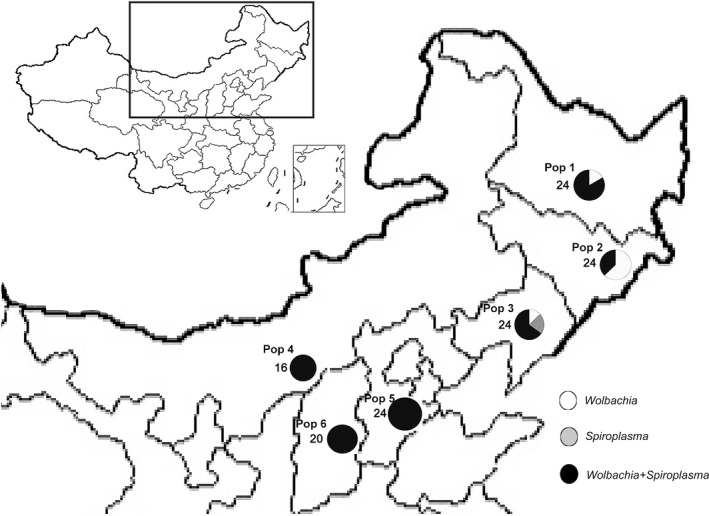
Infection frequencies of *Wolbachia* and *Spiroplasma* in six *Tetranychus truncatus* populations. Numbers indicate sample size and proportions of circles indicate infection frequencies. Individuals of Pop 1–6 were collected from Harbin Heilongjiang (N46.19–E125.0), Yanji Jilin (N43.13–E129.03), Shenyang Liaoning (N41.52–E123.22), Hohhot Inner Mongolia (N40.47–E111.47), Cangzhou Hebei (N38.18–E116.52) and Changzhi Shanxi (N35.55–E113.34), respectively, in August 2014

Adult *T. truncatus* were collected from six populations in northern China (Figure 1). Individual mites were either immediately analyzed in the laboratory to assess infection frequency (see below) or individual females were reared as isofemale lines on leaves of common bean (*Phaseolus vulgaris* L.) placed on a water‐saturated sponge mat in Petri dishes (dia. 9) at 25 ± 1°C, 60% humidity, and under L16–D8 conditions.

Subsequently, 16–24 field‐collected individual mites were screened for infection with *Wolbachia* and/or *Spiroplasma*. DNA was extracted from single mites using a genomic DNA extraction kit (TaKaRa, Dalian, China). All DNA samples were first PCR‐screened for the mitochondrial gene *COI* as a control for quality (Navajas, Gutierrez, Lagnel, & Boursot, 1996). *Wolbachia* and *Spiroplasma* presence was detected using PCR amplifications of *wsp* and *16S rRNA*, respectively. *wsp* was amplified using the primers *wsp*F1 and *wsp*R1 (Baldo et al., 2006), and *16S rRNA* was amplified using the primers SpitsJ04 and SpitsN55 (Jaenike, Polak, Fiskin, Helou, & Minhas, 2007). In addition, to confirm the presence of other two reproductive endosymbionts, *Cardinium* and *Rickettsia*, in *T. truncates*. PCR amplifications were performed using primers Ch‐F and Ch‐R (Zchori‐Fein & Perlman, 2004), RICS741F and RCIT1197R (Davis, Ying, Brunner, Pantoja, & Ferwerda, 1998) that were widely used to detect *Cardinium* and *Rickettsia*, respectively. Each reaction was carried out on a Veriti machine (ABI Biosystems, USA) in a 25 μl volume containing 12.5 μl 2× Taq Master Mix (Vazyme Biotech, China), 0.5 μl primer (20 μmol/L each), and 1 μl of DNA extract. Positive and negative controls were included in PCRs.

### 2.2 Establishment of experimental *T. truncatus* strains

Experimental *T. truncates* strains with different infection patterns were established to determine the effects of endosymbiont on host biology. In this study, we firstly established four *T. truncates* strains: mites infect with both *Wolbachia* and *Spiroplasma* (designated as W+S+), *Wolbachia* only (W+S−), *Spiroplasma* only (W−S+), and no symbionts (W−S−). To eliminate the effects of host genetic background, the above‐mentioned four *T. truncatus* strains were derived from lines collected in Shenyang, Liaoning (Pop 3 in Figure 1). The original field females included three strains, W+S+, W+S−, and W‐S+. The W−S− strains were obtained by raising infected mites on the common bean placed on a cotton bed soaked in tetracycline solution (0.1%, w/v) for three generations. These lines were maintained in a mass‐rearing environment without antibiotics for approximately six generations to allow recovery from tetracycline treatment. For each of these lines, infection status was checked during the course of the experiments.

### 2.3 Crossing experiments

**Figure 2:**
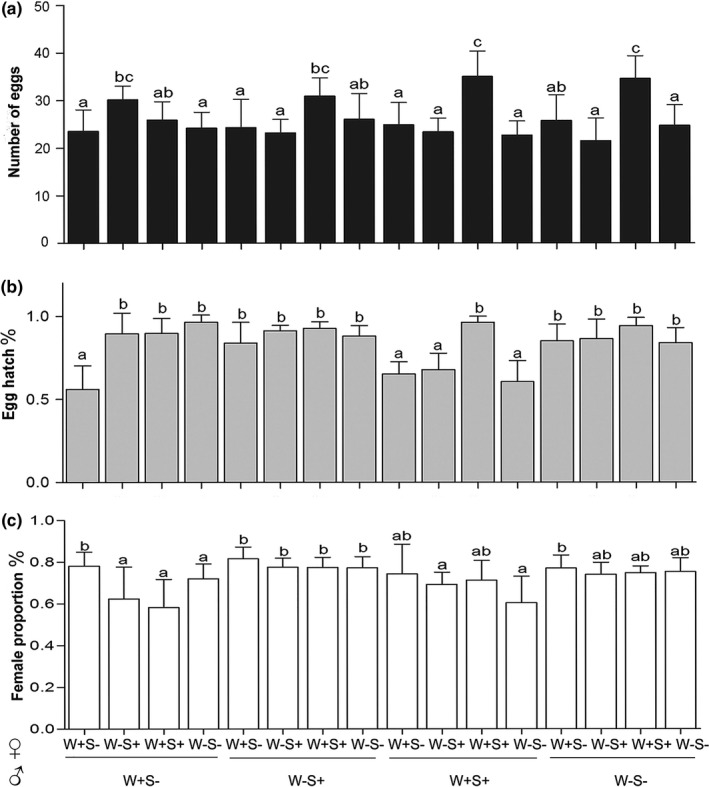
Results of crosses between different *Tetranychus truncatus* strains. Number of eggs (a), egg hatch percent (b), and female proportion of offspring (c) are shown. Results are mean ± *SEM*, and a and b represent statistically different groups (Tukey‐HSD test, *p *< .05)

To test whether *Wolbachia* and *Spiroplasma* cooperate to cause cytoplasmic incompatibility (CI) in *T. truncatus*, crossing experiments were performed (listed as male × female, Figure 2). Single females in the teleiochrysalis stage (the last developmental stage before adult emergence) were placed with a 1‐day‐old adult virgin male from the same culture on the same leaf disk. Each cross used 16 leaf disks. Males were discarded 2 days after the females reached adulthood. The mated females were allowed to oviposit for 5 days. The eggs on the leaf disks were checked daily to determine hatchability and the sex ratio (% females). Prior to analyses, data were first tested for normality (Kolmogorov–Smirnov test, SPSS 17.0) and homogeneity of group variances (Levene's test, SPSS 17.0). Which possible, square root, logarithmic or arcsine transformations were performed to attain normality and homogeneity of variance. In this study, log transformation was used for the number of eggs laid per female, and arcsine square root transformation was used for egg hatchability and sex ratio. Data were then analyzed with one‐way analysis of variance (ANOVA), and the means were compared using the Tukey HSD test (SPSS 17.0).

The male‐killing phenotype of *Spiroplasma* in arthropods results in the production of female‐biased offspring sex ratios. Here, the sex ratio data of the four *T. truncatus* strains were analyzed to determine if male‐killing occurred.

### 2.4 Survival assay

To test for potential effects of symbionts on host survival, we measured the survival of different *T. truncatus* strains by placing eight virgin females and eight virgin males from the same strains on the same leaf. Three leaves were used for each line, and females were transferred to fresh leaves every 3 days. The number of dead females was recorded daily. Survivor curves for individual hosts were compared using the Kaplan–Meier method and log‐rank test (Dobson, Rattanadechakul, & Marsland, 2004).

### 2.5 Development assay

The effect of symbionts on mite development was assessed. Thirty virgin and 30 mated females (which produce male and female offspring, respectively) were placed on a leaf disk and allowed to lay eggs for 8 hr. The eggs were individually moved to new small leaf disks. The small disks were monitored every 8 hr, and the stage of mite was recorded until adulthood. The development time of every stage was calculated. Log transformation was used for the total development time of each mite line, and differences were analyzed using the Mann–Whitney test (SPSS 17.0).

### 2.6 *Wolbachia* and *Spiroplasma* densities

The *Wolbachia* and *Spiroplasma* densities in individual mites were estimated by real‐time quantitative PCR (QPCR). QPCR was carried out with the ABI PRISM 7300 Sequence Detection System (Applied Biosystems). We used primers designed to amplify a 141‐bp fragment of *wsp* from *Wolbachia* (wQF1, 5′‐GAGCAGCGAATGTAAGCAATC‐3′, and wQR1, 5′‐AATAACGAGCACCAGCATAAAG‐3′) and a 141‐bp fragment of *16S rRNA* from *Spiroplasma* (sQF1, 5′‐TGTAGTTCTCAGGGATTGTTTTCTC‐3′, and sQR1, 5′‐CGCTTCCACCATCGCTCTT‐3′). The PCR products of primers specific for *wsp* for *Wolbachia* and *16S rRNA* for *Spiroplasma* were amplified by conventional PCR; then, the PCR products were purified using the AxyPrep TM DNA Gel Extraction kit (AXYGEN) and cloned into a pEASY‐T1 vector (TransGen Biotech, Beijing, China). A series of DNA standards prepared from plasmid DNA was used, and standard curves were plotted using a 10‐fold dilution series from 10^4^ to 10^8^ copy numbers. Ct values in each dilution were measured by QPCR to generate the standard curves for *wsp* and *16S rRNA*. The 20 μl reaction mixture consisted of 10 μl 2× SYBR Premix Ex Taq (Applied Biosystems), 0.4 μl 10 mmol/L of each primer, 0.4 μl 50× ROX Reference Dye, 2 μl DNA template, and 6.8 μl H_2_O in single wells of a 96‐well plate (PE Applied Biosystems). The QPCR cycling conditions included one cycle (10 s at 95°C) followed by 40 cycles (5 s at 95°C and 31 s at 60°C), and finally one cycle (15 s at 95°C, 1 min at 60°C and 15 s at 95°C).

Eight each of singly and doubly infected females of 2‐, 4‐, 6‐ 8‐, and 12‐days‐old were collected. DNA of individual mites was extracted as described above. Three replicates were run and averaged for each DNA sample. Negative controls were included in all amplification reactions. From the slopes, a high amplification efficiency of 0.95 was determined for both *wsp* and *16S rRNA* in the investigated range. The number of molecules in all samples is determined from the threshold cycles in the PCR based on a standard curve. Statistical analysis was performed using the Mann–Whitney *U* test.

## 3 RESULTS

### 3.1 Double infections of *Wolbachia* and *Spiroplasma* are frequent in *T. truncatus*

Among 132 wild‐caught adult mites that were collected from six sites, 16.7% and 3.8% were infected with only *Wolbachia* or *Spiroplasma*, respectively, and 79.5% were infected with both symbionts. Double infections with high prevalence were present in all screened populations (Figure 1). The infections of *Cardinium* and *Rickettsia* were not detected in these examined samples.

Sequencing of *Wolbachia wsp* identified two haplotypes that differed by one base (*w*Tru1 in Pop 2, 4, 5, and 6; *w*Tru5 in Pop 1 and 3). However, identical *Spiroplasma 16S rRNA* sequences were observed among infected individuals.

### 3.2 *Wolbachia* and *Spiroplasma* have diverse effects on host reproduction

To clarify the effects of *Wolbachia* and *Spiroplasma* on host reproduction, egg number, egg hatchability, and offspring sex ratio of the 16 crosses were compared (Figure 2 A, B, C). Among the four crosses in which we tested possible *Wolbachia* CI effects, the egg hatchability of the predicted compatible crosses (W+S− × W+S−) was significantly lower than that in other crosses, whereas the female proportion was significantly higher in this cross. Doubly infected males appeared to induce CI, which was inferred based on significant reductions of egg hatchability and female proportion of offspring in the predicted incompatible crosses. However, no significant effects were found for other crosses in which singly *Spiroplasma*‐infected males and *Spiroplasma*‐uninfected males were investigated. In addition, nonsignificant differences in female proportion of the four strains indicated that *Spiroplasma* does not cause male killing in *T. truncatus*.

The effects of *Wolbachia* and *Spiroplasma* on host fecundity were also measured. The doubly infected females mostly showed the highest egg production, except for the crosses that involved the singly *Wolbachia*‐infected males, in which singly *Spiroplasma*‐infected females produced more eggs. In general, egg production of singly infected females and uninfected females did not significantly differ across all crosses.

### 3.3 *Wolbachia* and *Spiroplasma* do not affect host survival

**Figure 3:**
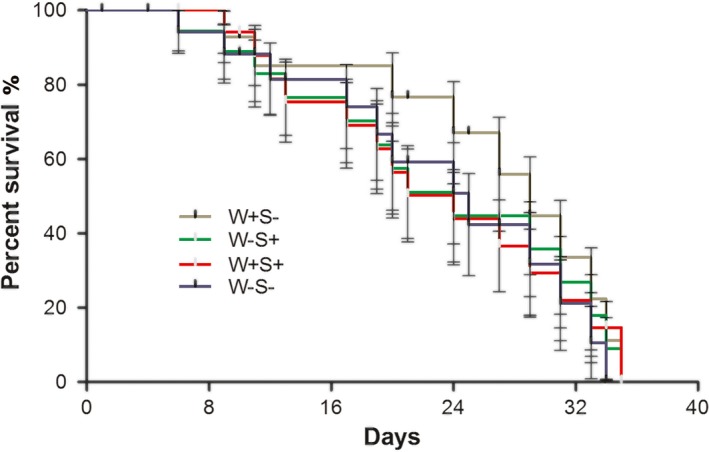
Comparison of *Wolbachia* and *Spiroplasma* effects on female *Tetranychus truncatus* survival. Survival curves for individual hosts were compared using the Kaplan–Meier method and log‐rank test

The longevities of four female lines were compared to discover the effects of infection. As shown in Figure 3, there was no significant effect of *Wolbachia* and *Spiroplasma* on female host survival (log‐rank test, *p *=* *.87).

### 3.4 Double infections can shorten host developmental rate

**Figure 4:**
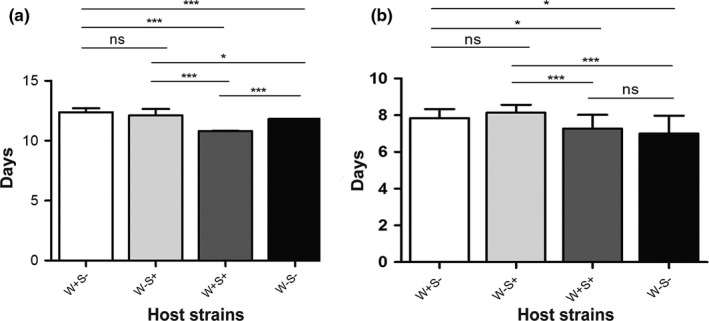
Development times of different female (a) and male (b) *Tetranychus truncatus* strains. Differences were analyzed using Mann–Whitney test (*p <* *.05; ***p <* *.001; NS, not significant)

The development times of females and males from different strains are presented in Figure 4A,B, respectively. Doubly infected females reached adulthood (10.8 ± 0.004) significantly earlier than singly infected females by 1–2 days (W+S−: 12.38 ± 0.13, W−S+: 12.11 ± 0.13) and uninfected females (11.81 ± 0.003) (*p *<* *.0001). Additionally, time to adulthood was significantly shorter in uninfected than singly infected females (*p *<* *.05), and the latter two had no significant difference (*p *=* *.97). Similarly, doubly infected (7.27 ± 0.19) and uninfected males (6.99 ± 0.25) developed faster than singly infected males (W+S−: 7.83 ± 0.15, W−S+: 8.13 ± 0.11) (*p *<* *.05), whereas no significant difference was detected between doubly infected and uninfected males (*p *=* *.41), singly *Wolbachia*‐ and singly *Spiroplasma*‐infected males (*p *=* *.31).

### 3.5 *Wolbachia* and *Spiroplasma* densities are higher in singly than doubly infected females

**Figure 5:**
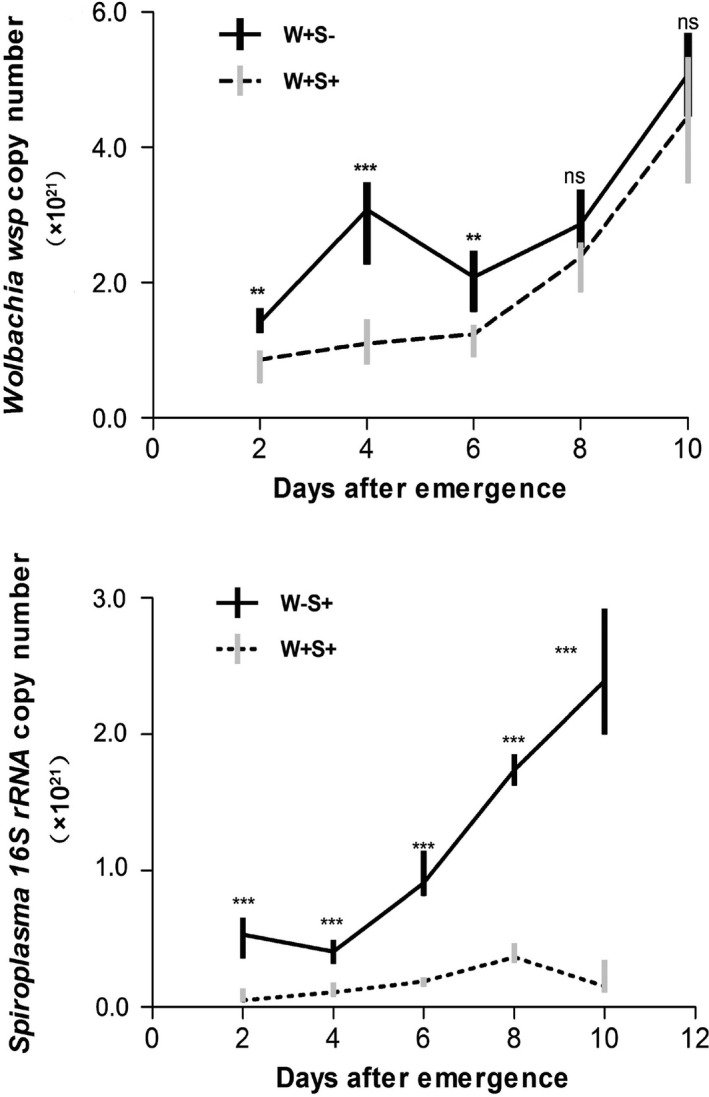
Density dynamics of *Wolbachia* and *Spiroplasma* during host development in singly and doubly infected female *Tetranychus truncatus*. Copy numbers per ml were determined by quantitative PCR. Asterisks indicate statistically significant differences (Mann–Whitney *U* test, *p < .05; **p < .01; ***p < .001; NS, not significant)

The *Wolbachia* density steadily increased (except for 2‐day‐old to 4‐day‐old adults of the singly infected strain) as adult females developed in both strains. At three developmental stages, 2, 4, and 6 days after emergence, the *Wolbachia* densities in singly infected females were significantly higher in doubly infected females (Figure 5A, *p* < .01). A similar pattern was observed for *Spiroplasma*, which revealed that *Spiroplasma* density dynamics were significantly affected by coinfecting *Wolbachia* (Figure 5B, *p* < .001).

## 4 DISCUSSION

In this study, we found that double infections of *Wolbachia* and *Spiroplasma* within the same individuals are frequent in natural *T. truncatus* populations. Among the six examined populations, 79.5% of mites were doubly infected, and double infection was fixed in three populations. *Wolbachia* and *Spiroplasma* are both maternally transmitted symbionts and act as reproductive manipulators in many arthropods. Symbiont infection frequency is theoretically determined by several factors, including reproductive manipulation, infection costs, vertical transmission efficiency, and horizontal transmission rate (Hoffmann, Hercus, & Dagher, 1998; Werren, 1997). In this respect, empirical studies have revealed that facultative symbionts can spread quickly within the host population when they provide a large fitness benefit at no or relatively little cost (Himler et al., 2011; Jaenike et al., 2010; Oliver, Campos, Moran, & Hunter, 2008). In such cases, reproductive manipulations and/or providing fitness benefits of double infections would be predicted in *T. truncatus*.

*Wolbachia*‐induced CI phenotypes have been observed in some *Tetranychus* species (Gotoh, Noda, & Hong, 2003; Zhao, Chen, Ge, Gotoh, & Hong, 2013; Zhu et al., 2012). However, there was no evidence of *Wolbachia*‐mediated CI in *T. truncatus*, which is consistent with a previous finding (Zhao, Chen, et al., 2013). Singly *Wolbachia*‐infected females may suffer fitness costs, such as lower hatch percent, but they also have a higher female offspring proportion. *Wolbachia* strains that infect *T. truncatus* are phylogenetically diverse (Zhang et al., 2013), and non‐CI *Wolbachia* were present in this study. Some likely reasons have been explained for the lack of CI (Ros & Breeuwer, 2009). Although *Spiroplasma* carried by *T. truncatus* has a closer phylogenetic relationship with the male‐killing strain in small brown planthopper, *Laodelphax striatellus* (Sanada‐Morimura et al., 2013), it is not a male killer, because *Spiroplasma* did not cause significant differences in offspring sex ratio, and its incidence in male hosts was relatively high.

It is worth noting that doubly infected males were incompatible with both singly infected and uninfected females, which was inferred based on significant reduction in the egg hatchability. The female offspring proportions of uninfected females were significantly lower than those of other strains, and symbionts carried by females might have rescue effects on offspring sex ratio. These observations suggested that double infections of *Wolbachia* and *Spiroplasma* can induce a CI‐like phenotype in *T. truncates*, and this phenotype is only associated with double infections. By comparison, the CI phenotype is different from the reports of double infections of *Wolbachia‐* and *Cardinium*‐induced CI in *T. truncatus* (Zhao, Chen, et al., 2013), *T. piercei* (Zhu et al., 2012), and *Bryobia sarothamni* (Ros & Breeuwer, 2009), in which double infections induced strong CI and single infections (*Wolbachia* or *Cardinium*) also induced CI. Together, we infer that interactions between *Wolbachia* and *Spiroplasma* resulted in CI induction, and the underlying mechanism merits further study.

Symbionts' complex effects on their hosts result from long‐term interactions and coevolution. Symbionts may manipulate their host physiological process and affect their fitness traits. *w*MelPop *Wolbachia* is well known for shortening the life of its *Drosophila* host (Min & Benzer, 1997). In *Drosophila hydei*,* Spiroplasma* showed enhanced survival when attacked by *Leptopilina heterotoma* wasps (Xie, Vilchez, & Mateos, 2010). Negative effects of symbionts on host survival were also observed (Costopoulos, Kovacs, Kamins, & Gerardo, 2014).

In this study, symbionts did not influence *T. truncatus* survival, but significantly regulated female fecundity. Relative to singly infected and uninfected females, doubly infected females produced more eggs. Fecundity advantage provided by symbionts is not surprising, and other studies report a similar phenomenon (Dobson et al., 2004; Himler et al., 2011; Zug & Hammerstein, 2015). These findings are concordant with a hypothesis that vertically transmitted symbionts cause their hosts to produce more infected daughters than are produced by uninfected females to facilitate their own spread (Werren & O'Neill, 1997). The infection density of symbionts is among the most important factors that influence their biological effects, such as intensity of reproductive phenotypes, level of fitness effects, and fidelity of vertical transmission. Oliver et al. (2006) proposed that over‐proliferation of symbionts may be harmful to the aphid host. In *T. truncatus*, the high density of symbionts may result in fecundity cost to singly infected females. Additionally, QPCR estimates also indicated that numbers of each of the two symbionts in singly infected females are significantly higher than those in doubly infected females.

In addition, double infections appeared to enhance female and male mites' developmental speed. Whatever the mechanism, it is evident that more generations will be produced within the doubly infected mite strains under certain circumstances. As a result, *T. truncatus* occurrence will directly benefit from these manipulations, and such manipulations will also maintain higher frequencies of double infection in nature. Similarly, Himler et al. (2011) found that *Rickettsia*‐infected whiteflies developed faster, and this observed increased performance can partly be explained by the rapid spread of *Rickettsia* across the southwestern United States within 8 years. Moreover, Jaenike et al. (2010) found that the frequent positive association between *Wolbachia* and *Spiroplasma* within *D. neotestacea* is a consequence of emerging symbiotic mutualism. Together, these observations strongly highlight symbionts' substantial influences on their hosts' ecology and population dynamics. Furthermore, these impacts are often influenced by symbionts (strains or infection patterns) and hosts (genetic background). For example, the fitness benefits to *T. truncatu*s are mainly associated with double infections, which raises the possibility that *Wolbachia* and *Spiroplasma* coordinately regulate host biological traits.

When several symbionts are simultaneously present within the same host, symbiont–symbiont interactions can take place and substantially affect infection densities. The symbionts may compete for available resources and space in the host body or they may share the resources and habitats by regulating their own exploitation so as not to damage the whole symbiotic system. Alternatively, symbiont infection density is governed by symbiont genotype, host genotype, and environment. For example, Goto, Anbutsu, and Fukatsu (2006) revealed asymmetrical interactions between *Wolbachia* and *Spiroplasma* endosymbionts that coexist in the same insect host, *D. melanogaster*. In this study, the QPCR result provided evidence of a competitive relationship between *Wolbachia* and *Spiroplasma* in doubly infected females of *T. truncatus*. The two symbionts are maternally transmitted and mostly located in the ovary; thus, their expansions are limited by resources and space in cases of co‐infection.

In summary, double infections of *Wolbachia* and *Spiroplasma* are beneficial to their mite host by inducing CI, and increasing fecundity and developmental rate. These benefits can help maintain stable double infections and also drive *T. truncatus* outbreaks in combination with other environmental factors.

## AUTHOR CONTRIBUTIONS

YKZ and XYH conceived and designed experiments. YKZ, KY, YXZ performed the experiments. YKZ and KY analyzed the data. YKZ and XYH wrote the paper. All authors read and approved the final manuscript.

## CONFLICT OF INTEREST

None declared.

## References

[ece33784-bib-0002] Baldo, L. , Hotopp, J. C. D. , Jolley, K. A. , Bordenstein, S. R. , Biber, S. A. , Choudhury, R. R. , … Werren, J. H. (2006). Multilocus sequence typing system for the endosymbiont *Wolbachia pipientis* . Applied and Environmental Microbiology, 72(11), 7098–7110. https://doi.org/10.1128/AEM.00731-06 16936055 10.1128/AEM.00731-06 PMC1636189

[ece33784-bib-0003] Baumann, P. (2005). Biology of bacteriocyte‐associated endosymbionts of plant sap‐sucking insects. Annual Review of Microbiology, 59, 155–189. https://doi.org/10.1146/annurev.micro.59.030804.121041 10.1146/annurev.micro.59.030804.121041 16153167

[ece33784-bib-0004] Bolland, H. R. , Gutierrez, J. , & Flechtmann, C. H. W. (1998). World catalogue of the spider mite family (Acari: Tetranychidae). Leiden, The Netherlands: Brill Academic press.

[ece33784-bib-0005] Cisak, E. , Wójcik‐Fatla, A. , Zając, V. , Sawczyn, A. , Sroka, J. , & Dutkiewicz, J. (2015). *Spiroplasma*‐an emerging arthropod‐borne pathogen? Annals of Agricultural and Environmental Medicine, 22(4), 589–593. https://doi.org/10.5604/12321966.1185758 26706960 10.5604/12321966.1185758

[ece33784-bib-0006] Costopoulos, K. , Kovacs, J. L. , Kamins, A. , & Gerardo, N. M. (2014). Aphid facultative symbionts reduce survival of the predator lady beetle *Hippodamia convergens* . BMC Ecology, 14(1), 5 https://doi.org/10.1186/1472-6785-14-5 24555501 10.1186/1472-6785-14-5 PMC3936903

[ece33784-bib-0007] Davis, M. J. , Ying, Z. , Brunner, B. R. , Pantoja, A. , & Ferwerda, F. H. (1998). *Rickettsial* relative associated with papaya bunchy top disease. Current Microbiology, 26, 80–84. https://doi.org/10.1007/s002849900283 10.1007/s002849900283 9425244

[ece33784-bib-0008] Dobson, S. L. , Rattanadechakul, W. , & Marsland, E. J. (2004). Fitness advantage and cytoplasmic incompatibility in *Wolbachia* single‐ and superinfected *Aedes albopictus* . Heredity, 93(2), 135–142. https://doi.org/10.1038/sj.hdy.6800458 15127087 10.1038/sj.hdy.6800458

[ece33784-bib-0009] Duron, O. , Bouchon, D. , Boutin, S. , Bellamy, L. , Zhou, L. , Engelstädter, J. , … Hurst, G. D. (2008). The diversity of reproductive parasites among arthropods: *Wolbachia* do not walk alone. BMC Biology, 6(1), 27 https://doi.org/10.1186/1741-7007-6-27 18577218 10.1186/1741-7007-6-27 PMC2492848

[ece33784-bib-0010] Enigl, M. , & Schausberger, P. (2007). Incidence of the endosymbionts *Wolbachia*,* Cardinium* and *Spiroplasma* in phytoseiid mites and associated prey. Experimental and Applied Acarology, 42, 75–85. https://doi.org/10.1007/s10493-007-9080-3 17554631 10.1007/s10493-007-9080-3

[ece33784-bib-0011] Goodacre, S. L. , Martin, O. Y. , Thomas, C. F. G. , & Hewitt, G. M. (2006). *Wolbachia* and other endosymbiont infections in spiders. Molecular Ecology, 15, 517–527. https://doi.org/10.1111/j.1365-294X.2005.02802.x 16448417 10.1111/j.1365-294X.2005.02802.x

[ece33784-bib-0012] Goto, S. , Anbutsu, H. , & Fukatsu, T. (2006). Asymmetrical interactions between *Wolbachia* and *Spiroplasma* endosymbionts coexisting in the same insect host. Applied and Environmental Microbiology, 72(7), 4805–4810. https://doi.org/10.1128/AEM.00416-06 16820474 10.1128/AEM.00416-06 PMC1489378

[ece33784-bib-0013] Gotoh, T. , Noda, H. , & Hong, X. Y. (2003). *Wolbachia* distribution and cytoplasmic incompatibility based on a survey of 42 spider mite species (Acari: Tetranychidae) in Japan. Heredity, 91, 208–216. https://doi.org/10.1038/sj.hdy.6800329 12939620 10.1038/sj.hdy.6800329

[ece33784-bib-0014] Gündüz, E. A. , & Douglas, A. E. (2009). Symbiotic bacteria enable insect to use a nutritionally inadequate diet. Proceedings of the Royal Society of London B: Biological Sciences, 276(1658), 987–991. 10.1098/rspb.2008.1476 PMC2664372 19129128

[ece33784-bib-0015] Hedges, L. M. , Brownlie, J. C. , O'Neill, S. L. , & Johnson, K. N. (2008). *Wolbachia* and virus protection in insects. Science, 322(5902), 702 https://doi.org/10.1126/science.1162418 18974344 10.1126/science.1162418

[ece33784-bib-0016] Himler, A. G. , Adachi‐Hagimori, T. , Bergen, J. E. , Kozuch, A. , Kelly, S. E. , Tabashnik, B. E. , … Hunter, M. S. (2011). Rapid spread of a bacterial symbiont in an invasive whitefly is driven by fitness benefits and female bias. Science, 332(6026), 254–256. https://doi.org/10.1126/science.1199410 21474763 10.1126/science.1199410

[ece33784-bib-0017] Hoffmann, A. A. , Hercus, M. , & Dagher, H. (1998). Population dynamics of the *Wolbachia* infection causing cytoplasmic incompatibility in *Drosophila melanogaster* . Genetics, 148, 221–231. 9475734 10.1093/genetics/148.1.221 PMC1459765

[ece33784-bib-0018] Hosokawa, T. , Koga, R. , Kikuchi, Y. , Meng, X. Y. , & Fukatsu, T. (2010). *Wolbachia* as a bacteriocyte‐associated nutritional mutualist. Proceedings of the National Academy of Sciences of the United States of America, 107(2), 769–774. https://doi.org/10.1073/pnas.0911476107 20080750 10.1073/pnas.0911476107 PMC2818902

[ece33784-bib-0019] Hurst, G. D. , & Darby, A. C. (2009). The inherited microbiota of arthropods, and their importance in understanding resistance and immunity. Insect Infection and Immunity: Evolution, Ecology, and Mechanisms, 25, 119–136. https://doi.org/10.1093/acprof:oso/9780199551354.001.0001

[ece33784-bib-0020] Jaenike, J. , Polak, M. , Fiskin, A. , Helou, M. , & Minhas, M. (2007). Interspecific transmission of endosymbiotic *Spiroplasma* by mites. Biology Letter, 3, 23–25. https://doi.org/10.1098/rsbl.2006.0577 10.1098/rsbl.2006.0577 PMC2373825 17443956

[ece33784-bib-0021] Jaenike, J. , Stahlhut, J. K. , Boelio, L. M. , & Unckless, R. L. (2010). Association between *Wolbachia* and *Spiroplasma* within *Drosophila neotestacea*: An emerging symbiotic mutualism? Molecular Ecology, 19, 414–425. https://doi.org/10.1111/j.1365-294X.2009.04448.x 20002580 10.1111/j.1365-294X.2009.04448.x

[ece33784-bib-0022] Jiggins, F. M. , Hurst, G. D. D. , Jiggins, C. D. , VD Schulenburg, J. H. G. , & Majerus, M. E. N. (2000). The butterfly *Danaus chrysippus* is infected by a male‐killing *Spiroplasma* bacterium. Parasitology, 120(05), 439–446. https://doi.org/10.1017/S0031182099005867 10840973 10.1017/s0031182099005867

[ece33784-bib-0023] Ma, W. J. , Vavre, F. , & Beukeboom, L. W. (2014). Manipulation of arthropod sex determination by endosymbionts: Diversity and molecular mechanisms. Sexual Development, 8(1–3), 59–73. https://doi.org/10.1159/000357024 24355929 10.1159/000357024

[ece33784-bib-0024] Min, K. T. , & Benzer, S. (1997). *Wolbachia*, normally a symbiont of *Drosophila*, can be virulent, causing degeneration and early death. Proceedings of the National Academy of Sciences of the United States of America, 94(20), 10792–10796. https://doi.org/10.1073/pnas.94.20.10792 9380712 10.1073/pnas.94.20.10792 PMC23488

[ece33784-bib-0501] Migeon, A. , & Dorkeld, F. (2006–2017). Spider mites web: A comprehensive database for the Tetranychidae. Retrieved from http://www.montpellier.inra.fr/CBGP/spmweb

[ece33784-bib-0025] Navajas, M. , Gutierrez, J. , Lagnel, J. , & Boursot, J. (1996). Mitochondrial cytochrome oxidase I in tetranychid mites: A comparison between molecular phylogeny and changes of morphological and life history traits. Bulletin of Entomology Research, 86, 407–417. https://doi.org/10.1017/S0007485300034994

[ece33784-bib-0502] Oliver, K. M. , Moran, N. A. , & Hunter, M. S. (2006). Costs and benefits of a superinfection of facultative symbionts in aphids. Proceedings of the Royal Society of London B: Biological Sciences, 273(1591), 1273–1280. 10.1098/rspb.2005.3436 PMC1560284 16720402

[ece33784-bib-0026] Oliver, K. M. , Campos, J. , Moran, N. A. , & Hunter, M. S. (2008). Population dynamics of defensive symbionts in aphids. Proceedings of the Royal Society of London B: Biological Sciences, 275(1632), 293–299. https://doi.org/10.1098/rspb.2007.1192 10.1098/rspb.2007.1192 PMC2593717 18029301

[ece33784-bib-0027] Ros, V. I. D. , & Breeuwer, J. A. J. (2009). The effects of, and interactions between, *Cardinium* and *Wolbachia* in the doubly infected spider mite *Bryobia sarothamni* . Heredity, 102, 413–422. https://doi.org/10.1038/hdy.2009.4 19223923 10.1038/hdy.2009.4

[ece33784-bib-0028] Sanada‐Morimura, S. , Matsumura, M. , & Noda, H. (2013). Male killing caused by a *Spiroplasma* symbiont in the small brown planthopper, *Laodelphax striatellus* . Journal of Heredity, 104(6), 821–829. https://doi.org/10.1093/jhered/est052 23975837 10.1093/jhered/est052

[ece33784-bib-0029] Shokal, U. , Yadav, S. , Atri, J. , Accetta, J. , Kenney, E. , Banks, K. , … Eleftherianos, I. (2016). Effects of co‐occurring *Wolbachia* and *Spiroplasma* endosymbionts on the *Drosophila* immune response against insect pathogenic and non‐pathogenic bacteria. BMC Microbiology, 16(1), 16 https://doi.org/10.1186/s12866-016-0634-6 26862076 10.1186/s12866-016-0634-6 PMC4746768

[ece33784-bib-0030] Tinsley, M. C. , & Majerus, M. E. N. (2006). A new male‐killing parasitism: *Spiroplasma* bacteria infect the ladybird beetle *Anisosticta novemdecimpunctata* (Coleoptera: Coccinellidae). Parasitology, 132(06), 757–765. https://doi.org/10.1017/S0031182005009789 16454865 10.1017/S0031182005009789

[ece33784-bib-0031] Vorburger, C. , Gehrer, L. , & Rodriguez, P. (2010). A strain of the bacterial symbiont *Regiella insecticola* protects aphids against parasitoids. Biology Letters, 6(1), 109–111. 19776066 10.1098/rsbl.2009.0642 PMC2817266

[ece33784-bib-0032] Werren, J. H. (1997). Biology of *Wolbachia* . Annual Review of Entomology, 42(1), 587–609. https://doi.org/10.1146/annurev.ento.42.1.587 10.1146/annurev.ento.42.1.587 15012323

[ece33784-bib-0033] Werren, J. H. , Baldo, L. , & Clark, M. E. (2008). *Wolbachia*: Master manipulators of invertebrate biology. Nature Reviews Microbiology, 6(10), 741–751. https://doi.org/10.1038/nrmicro1969 18794912 10.1038/nrmicro1969

[ece33784-bib-0034] Werren, J. H. , & O'Neill, S. L. (1997). The evolution of heritable symbionts In O'Neill S. L., Hoffmann A. A., & Werren J. H. (Eds.), Influential passengers: Inherited microorganisms and arthropod reproduction (pp. 1–41). Oxford: Oxford University Press.

[ece33784-bib-0035] Williamson, D. L. , Sakaguchi, B. , Hackett, K. J. , Whitcomb, R. F. , Tully, J. G. , Carle, P. , & Henegar, R. B. (1999). *Spiroplasma poulsonii* sp. nov., a new species associated with male‐lethality in *Drosophila willistoni*, a neotropical species of fruit fly. International Journal of Systematic and Evolutionary Microbiology, 49(2), 611–618. 10.1099/00207713-49-2-611 10319483

[ece33784-bib-0036] Xie, J. , Vilchez, I. , & Mateos, M. (2010). *Spiroplasma*, bacteria enhance survival of, *Drosophila hydei*, attacked by the parasitic wasp, *Leptopilina heterotoma* . PLoS ONE, 5(8), 3733–3756. 10.1371/journal.pone.0012149 PMC2921349 20730104

[ece33784-bib-0037] Zchori‐Fein, E. , & Perlman, S. J. (2004). Distribution of the bacterial symbiont *Cardinium* in arthropods. Molecular Ecology, 13, 2009–2016. https://doi.org/10.1111/j.1365-294X.2004.02203.x 15189221 10.1111/j.1365-294X.2004.02203.x

[ece33784-bib-0038] Zhang, Y. K. , Chen, Y. T. , Yang, K. , Qiao, G. X. , & Hong, X. Y. (2016). Screening of spider mites (Acari: Tetranychidae) for reproductive endosymbionts reveals links between co‐infection and evolutionary history. Scientific Reports, 6, 27900 https://doi.org/10.1038/srep27900 27291078 10.1038/srep27900 PMC4904281

[ece33784-bib-0039] Zhang, Y. K. , Zhang, K. J. , Sun, J. T. , Yang, X. M. , Ge, C. , & Hong, X. Y. (2013). Diversity of *Wolbachia* in natural populations of spider mites (genus *Tetranychus*): Evidence for complex infection history and disequilibrium distribution. Microbial Ecology, 65, 731–739. https://doi.org/10.1007/s00248-013-0198-z 23429887 10.1007/s00248-013-0198-z

[ece33784-bib-0040] Zhao, D. X. , Chen, D. S. , Ge, C. , Gotoh, T. , & Hong, X. Y. (2013). Multiple infections with *Cardinium* and two strains of *Wolbachia* in the spider mite *Tetranychus phaselus* Ehara: Revealing new forces driving the spread of *Wolbachia* . PLoS ONE, 8, e54964 https://doi.org/10.1371/journal.pone.0054964 23355904 10.1371/journal.pone.0054964 PMC3552951

[ece33784-bib-0041] Zhao, D. X. , Zhang, X. F. , & Hong, X. Y. (2013). Host‐symbionts interactions in spider mite *Tetranychus truncatus* doubly infected with *Wolbachia* and *Cardinium* . Environmental Entomology, 42, 445–452. https://doi.org/10.1603/EN12354 23726053 10.1603/EN12354

[ece33784-bib-0042] Zhu, L. Y. , Zhang, K. J. , Zhang, Y. K. , Ge, C. , Gotoh, T. , & Hong, X. Y. (2012). *Wolbachia* strengthens *Cardinium*‐induced cytoplasmic incompatibility in the spider mite *Tetranychus piercei* McGregor. Current Microbiology, 65, 516–523. https://doi.org/10.1007/s00284-012-0190-8 22806335 10.1007/s00284-012-0190-8

[ece33784-bib-0043] Zug, R. , & Hammerstein, P. (2015). Bad guys turned nice? A critical assessment of *Wolbachia* mutualisms in arthropod hosts. Biological Reviews, 90(1), 89–111. https://doi.org/10.1111/brv.12098 24618033 10.1111/brv.12098

